# Quality of Life in Transitioned Trans Persons: A Retrospective Cross-Sectional Cohort Study

**DOI:** 10.1155/2018/8684625

**Published:** 2018-04-12

**Authors:** Lena Jellestad, Tiziana Jäggi, Salvatore Corbisiero, Dirk J. Schaefer, Josef Jenewein, Andres Schneeberger, Annette Kuhn, David Garcia Nuñez

**Affiliations:** ^1^Department of Psychiatry and Psychotherapy, University Hospital Zurich, University of Zurich, Rämistrasse 100, 8091 Zurich, Switzerland; ^2^Department of Psychology, University of Zurich, Binzmühlestrasse 14, 8050 Zurich, Switzerland; ^3^Division of Clinical Psychology and Psychiatry, University of Basel, Wilhelm Klein-Strasse 27, 4002 Basel, Switzerland; ^4^Department of Plastic, Reconstructive, Aesthetic and Hand Surgery, Basel University Hospital, University of Basel, Spitalstrasse 21, 4031 Basel, Switzerland; ^5^Psychiatric Services Graubünden, Loëstrasse 220, 7000 Chur, Switzerland; ^6^Department of Obstetrics and Gynecology, University Hospital Bern, Effingerstrasse 102, 3010 Bern, Switzerland; ^7^Center for Gender Variance, Basel University Hospital, University of Basel, Spitalstrasse 21, 4031 Basel, Switzerland

## Abstract

**Background:**

Medical gender-affirming interventions (GAI) are important in the transition process of many trans persons. The aim of this study was to examine the associations between GAI and quality of life (QoL) of transitioned trans individuals.

**Methods:**

143 trans persons were recruited from a multicenter outpatient Swiss population as well as a web-based survey. The QoL was assessed using the Short Form (36) Health Survey questionnaire (SF-36). Depressive symptoms were examined using the Short Form of the Center for Epidemiologic Studies-Depression Scale (ADS-K). Multiple interferential analyses and a regression analysis were performed.

**Results:**

Both transfeminine and transmasculine individuals reported a lower QoL compared to the general population. Within the trans group, nonbinary individuals showed the lowest QoL scores and significantly more depressive symptoms. A detailed analysis identified sociodemographic and transition-specific influencing factors.

**Conclusions:**

Medical GAI are associated with better mental wellbeing but even after successful medical transition, trans people remain a population at risk for low QoL and mental health, and the nonbinary group shows the greatest vulnerability.

## 1. Introduction

Gender incongruence (GI) is a condition in which the gender identity or gender expression of a person is discordant with their assigned sex characteristics. GI is often accompanied by clinically relevant psychological distress, then called gender dysphoria (GD) [1]. Individuals with a GI are usually referred to as trans persons. This umbrella term covers persons whose gender identity is the opposite of their assigned sex (transmen and transwomen). It also includes persons who place themselves between or outside the binary gender categories (nonbinary persons).

GD can present with a strong rejection of the anatomical characteristics—primarily the sexual features [1]. Furthermore, because of the ubiquitous social stigma that trans persons experience [2], GD can lead to negative self-image and mental health problems [3–5]. In particular, many trans individuals experience depressive episodes during their lifetime, which in the worst case are associated with suicidal behavior [3]. To overcome this feeling of GD, many trans persons seek medical help and undergo gender-affirming interventions (GAI) (e.g., sex hormonal treatment or gender-affirming surgery). Nowadays, it is generally accepted that both hormonal and surgical interventions can alleviate GD [6–8].

Previous evidence on the negative psychological and QoL effects of GAI is rare and is based on older studies [9, 10]. In contrast, the positive effects on psychological wellbeing and QoL in trans persons have been reported more frequently [7, 8, 11–17]. Several studies have shown that a medical transition can positively impact the social status of treated trans persons [18]. However, it is also known that various psychosocial factors such as old age [7], unemployment [7, 19], being single [7, 20], having a low level of education [7], and depressive disorders [12] can negatively influence QoL. Many GAI require long-term care and follow-up, which can have a negative impact on wellbeing [21]. Consequently, the QoL of transitioned trans persons remains low compared to the general population [16, 22].

The literature is unclear regarding potential gender differences within the trans population. While some authors found no considerable differences in QoL scores between transwomen and transmen [12, 23], others noted significant divergences. For example, Parola et al. reported that transwomen have a better QoL in different mental and physical domains than transmen [14]. In contrast, Motmans et al. [7] found lower QoL scores in transwomen compared to transmen. Similar conflicting evidence is found in studies on mental health comparisons between trans persons. While some studies demonstrated worse mental health in transwomen [24], others found no gender-related differences [25].

Furthermore, this previous outcome research mainly used binary assumptions about gender identities and focused on either transfeminine or transmasculine individuals. There is little research on gender nonbinary persons [26]. This situation is caused by many factors but is particularly related to the theoretical and methodological issues of earlier study designs [27]. However, this unique subpopulation of trans persons seems to be especially prone to stigmatization [2], poor self-rated health [28], and high rates of affective disorders [29].

Therefore, the aim of this present study was to examine the associations between GAI and QoL in transitioned trans individuals. In particular, we investigated potential psychological and social factors that could influence the wellbeing of trans persons. We emphasized a broader gender spectrum by including gender nonbinary persons. For the first time, this provides a simultaneous comparison between different transitioned trans subgroups in terms of QoL.

## 2. Methods

We performed a retrospective cross-sectional multicenter study on persons self-identified as being trans. The study was performed in collaboration with the Department of Plastic, Reconstructive, Aesthetic, and Hand Surgery at Basel University Hospital, the Department of Psychiatry and Psychotherapy of the University Hospital Zurich, the Department of Psychiatry and Psychotherapy of the University Hospital Bern, and the Psychiatric Services Hospitals of the Canton of Solothurn.

### 2.1. Sample

We applied two ways of recruitment. Trans persons who had formerly presented to the outpatient clinic of the collaborating hospitals were invited by post to participate in the study and could choose between a paper-based form and a web-based survey. To maximize the number of participants, recruiting was expanded to the larger Swiss trans community via advocacy groups. These participants could only use the web-based survey. Data was collected anonymously in both ways of recruitment. The inclusion criteria included a minimum age of 18 years, a good command of the German language, and self-identification of being transitioned, that is, participants defining their medical transition to be completed independent of the extent of medical affirming interventions performed. Here, we defined the latter by the absence of psychiatric treatment associated with gender dysphoria, a minimum of one year of hormonal treatment, and no planned surgical intervention for the upcoming year. The exclusion criterion was an unfinished medical transitioning process. The study was approved by the Ethics Committee of Basel.

### 2.2. Measurements

#### 2.2.1. Quality of Life

The Short Form (36) Health Survey questionnaire (SF-36) is a widely used self-reported questionnaire on QoL and health status which assesses four mental domains of health (vitality, social functioning, emotional role functioning, and mental wellbeing) and four physical domains of health (physical functioning, physical role functioning, bodily pain, and general health). The two global measures can be derived from all physical and all mental domains, respectively, and are referred to as the physical component summary (PCS) and the mental component summary (MCS). Answers were transformed via a standardized measure according to the predefined scoring algorithm to yield a total score ranging from zero to 100; higher values indicate a higher subjective quality of life. Cronbach's alpha is an indicator of internal consistency and yielded a high score of .87.

In addition to group comparisons, we performed a *t*-test group comparison of the mean scores of transfeminine and transmasculine individuals with mean scores of men and women in the German general population. The SF-36 German general population data were taken from the 1995 German population survey [30]. No standard values exist for nonbinary individuals.

#### 2.2.2. Depressive Symptoms

The Allgemeine Depressionsskala (ADS-K) is a validated German short form adaptation of the Center for Epidemiologic Studies Depressions Scale (CES-D), a screening instrument for depressive symptoms [31, 32]. It covers 15 items on affective, motivational, psychosocial, somatic, and cognitive symptoms. The total score ranges from zero to 45, with higher values indicating more severe depressive symptoms. A score ≥ 18 is the cut-off for clinically relevant depressive disorders [33]. For reliability analysis, Cronbach's alpha was calculated to assess the internal consistency of the ADS-K questionnaire in our sample. With a Cronbach's alpha value of .85, the internal consistency of the questionnaire is high and comparable with the reported value of *α* = .88–.95 in the ADS-K manual [31].

#### 2.2.3. Sociodemographic Characteristics

The sociodemographic survey was thematically divided into three categories: general, trans-specific, and transition-specific. In the general part, participants self-reported their age, current housing situation, relationship status, level of education, and current work situation. The trans-specific variables included gender assigned at birth and preferred gender label. The transition-specific variables included prior hormonal and surgical affirming interventions.

### 2.3. Data Analysis

We used SPSS version 22.0 for all statistical analysis. Frequency distributions were analyzed using chi-square tests. A set of *t*-tests and one-way ANOVA tests were calculated to compare the mean scores between gender groups. We performed multiple multivariate regression analyses to identify predictor variables to QoL. We considered two-tailed *p* values < 0.05 to be significant.

## 3. Results

### 3.1. Sample

We contacted 373 individuals from outpatient clinics via a written form and asked them to participate in the study; 66 completed questionnaires were returned (response rate of 18.0%). There were 201 web participants, of whom 77 individuals completed it. There was an inclusion rate of 38%. In total, 143 individuals completed the questionnaire and were included. In this study, we defined a transfeminine person as an individual self-identified as female with a male sex assigned at birth and a transmasculine person as an individual self-identified as male with a female sex assigned at birth; a gender nonbinary person is an individual self-identified in-between male and female gender or self-identified as no gender, independent of the assigned sex at birth. The majority of our sample was transfeminine (*n* = 77; 53.8%) followed by transmasculine (*n* = 41; 28.7%) and nonbinary gender (*n* = 25; 17.5%). Within the group of nonbinary individuals, 7 persons (28%) had a male sex assigned at birth and 17 persons (68%) had a female sex assigned at birth. One person stated to have an “other” sex assigned at birth.

### 3.2. Sociodemographic Characteristics

Participants' age ranged from 18 to 75 years (Table 1). Transfeminine individuals were older than transmasculine individuals and nonbinary individuals; the difference between transfeminine and transmasculine individuals was significant (*p* < 0.01). There was no significant difference in the level of education, relationship status, work, and housing situation between groups. Post hoc test of the variable relationship status provided no significant differences between groups (data not shown).

### 3.3. Transition-Specific Characteristics

Compared to transmasculine individuals, transfeminine individuals were significantly (*p* < 0.01) older at coming out and proceeded faster into the transition process (transmasculine individuals after 4.39 years versus transfeminine individuals after 0.32 years) (Table 2). On one hand, nonbinary individuals showed distinctly different traits representing a population “in-between” transfeminine and transmasculine individuals concerning distribution of age at coming out and first steps into transition. On the other hand, by making the first steps of medical transition before actually coming out, the nonbinary group significantly differed from the binary trans people. The same dichotomous pattern between binary and nonbinary trans persons was also found in current hormone treatment and the overall demand for gender-affirming surgical interventions. Within the binary group, an analysis of the surgical interventions performed showed gender-specific patterns. While mastectomy was very common (92.6%) and phalloplasty was less used (29.2%) in men, breast augmentation (58.4%) and vaginoplasty (64.9%) were reported in a more similar frequency in women.

### 3.4. ADS-K

Gender had a significant effect on ADS-K scores between groups (*F* = 5.98; *p* ≤ 0.01). Post hoc tests demonstrate that gender nonbinary individuals rated significantly higher (M (SD): 18.04 (10.17)) compared to transfeminine (M (SD): 10.76 (7.80)) and transmasculine (M (SD): 12.59 (10.16)) individuals. This suggests higher affective distress in this population. The difference between transfeminine and transmasculine individuals was statistically nonsignificant.

### 3.5. SF-36

Transfeminine individuals scored worse than transmasculine individuals did in both the somatic and mental domains (Table 3), except in the domains general health and mental health. However, these differences were not significant. Nonbinary individuals performed worse than the other two groups in seven out of eight domains with significant results in the domains physical role, general health, vitality, mental health, and emotional role. In addition, the mental component summary (MCS) score of the nonbinary persons was significantly lower than that in the other groups.

In comparison to the German general population, both transfeminine and transmasculine individuals scored lower in the mental component summary score (MCS) of the SF-36 (Table 4). In transfeminine individuals, the mental domains social functioning, emotional role, and the MCS showed significantly reduced values compared to the general female population. Transmasculine individuals rated the mental domains vitality and social functioning significantly lower than the general male population.

The physical component summary scores were significantly higher in both trans groups compared to the general population. Within all the physical domains, QoL was rated higher compared to the general population in both transfeminine and transmasculine individuals. Among these, scores in the domain physical functioning were significant in both transfeminine and transmasculine individuals compared to the general population. Transfeminine individuals also rated general health significantly higher than the general population.

### 3.6. Interferential Analysis

Factors that have a significant negative influence on quality of life in the global measures include young age, having an “other” relationship status (different from being in a relationship and being single), being unemployed, and having an “other” work situation (different from being employed or unemployed) (Table 5).

Medical transition measures such as hormone therapy and gender-confirmation surgery have strong implications on the mental wellbeing and self-rated quality of life depicted by significantly reduced MCS values (Table 6).

### 3.7. Multiple Regression Analysis

A block-wise multiple regression analysis was performed to estimate the relationships between MCS and gender, medical affirming interventions, work situation, and relationship status (Table 7). The full model explained 20% of the variance (*R*^2^ = .196,  *F*(8/113 = 4.68), and *p* < 0.001) with only gender and employment having significant regression weights. Gender contributed more to the model than employment.

## 4. Discussion

This study expanded research into the associations between gender-affirming interventions (GAI) and quality of life (QoL) of transitioned trans persons. Compared to the general population, these findings indicate poor quality of life in trans persons who had performed those medical interventions that they deemed necessary for their transition. However, not all trans persons are affected to the same extent by this situation: among trans people, the nonbinary group scored significantly worse on QoL and depressive symptoms than the transfeminine and transmasculine participants. First interferential analysis identified different sociodemographic and transition-specific influencing factors on the QoL of transitioned trans persons. Multiple regression analysis, however, failed to validate a relevant correlation between GAI and QoL. The model only confirmed a significant correlation between gender, work situation, and QoL.

### 4.1. Comparison to the General Population

To the best of our knowledge, this is the first study to evaluate QoL in both transfeminine and transmasculine individuals compared to the general population. In agreement with previous findings in transwomen [13] and transmen [7, 16, 20], the participants showed worse scores than the general population in virtually all measurement ranges of mental QoL. Of particular note is the significant difference in the social functioning domain of the SF-36, which was found in both genders. A possible explanation for this difference might be the still prevalent gender-binary model of western societies which reinforces the social stigma of trans persons [34]. These experiences of stigmatization constitute a specific so-called minority stress, which in turn affects the social functioning, the physical and mental health, and ultimately the QoL of trans persons [35].

A different picture is provided with regard to the physical dimensions of the QoL. We found a significantly increased physical component summary (PCS) score in both transfeminine and transmasculine participants compared to the general population. In addition, both groups showed a significant difference in the “physical functioning” domain. This concurs with observations that found physical-related QoL to be higher in transwomen [11, 13]. Ainsworth and Spiegel [11] suggested higher rates of chronic and acute disease in the general population as an underlying cause for this discrepancy. Following this argument, another possible explanation for this finding might be a selection bias of physically healthier individuals in our sample as most of the participants underwent surgical GAI, which are generally only amenable to largely physically healthy patients. Other studies, however, described no statistically significant differences in physical functioning compared to the general population [7, 16, 20].

### 4.2. Comparison between Transfeminine and Transmasculine Persons

In line with the findings of Auer et al. [23] and Gorin-Lazard et al. [12, 36], we found no significant differences in QoL between transfeminine and transmasculine persons. However, this contrasts with previous observations showing physical functioning and general health domains to be better in transmen than in transwomen and bodily pain to be better in transwomen than in transmen [7]. The latter result coincides with the clinical experience that masculinizing surgical procedures in the genital region are more complex to perform, are associated with more complications, and cause more discomfort. The fact that in our sample much less transmasculine than transfeminine persons have undergone a genital-confirming operation suggests that some transmen have decided against carrying out a phalloplasty in order not to endanger their own QoL. In this sense, the small number (12 cases) could have contributed to minimizing this gender-specific difference.

At the same time, there are studies (e.g., Parola et al. [14]) suggesting that the transmasculine population has better social functioning and mental health than the transfeminine population. In literature, this difference is attributed to the different ways in which society deals with transwomen and transmen. While transwomen must perform a good, if not perfect, (cis) passing, society gives transmen more freedom in this regard [37]. The fact that we have not established this gender difference cannot be explained with complete certainty. Nevertheless, it can be assumed that some of the transient transwomen develop mechanisms over time such that these stigmatizing situations do not affect their QoL.

### 4.3. Nonbinary Trans Persons

Our results show considerably different characteristics within the group of trans participants. While we did not detect significant differences in the QoL between transfeminine and transmasculine participants, persons with a nonbinary gender identity presented the lowest rates of wellbeing. They showed significantly worse values in five of eight QoL domains as well as in the mental component summary (MCS) compared to both binary groups. Moreover, a nonbinary gender identity was associated with significantly more depressive symptoms compared to the transfeminine and transmasculine groups.

The reasons for these clear group differences should be determined on different levels. First, nonbinary persons reported specific needs regarding medical GAI. Thus, approximately half of this group decided not to seek medical treatment. This diversity in terms of the GAI undertaken has been confirmed by other studies [38]. The comparatively worse QoL of the nonbinary participants could therefore be related to the lack of a standardized treatment and accordingly suitable GAI, which cover the specific needs of this group [39]. Second, the nonbinary group most clearly questions the binary gender norm that exists in western societies. As a result, nonbinary individuals are more likely to be confronted with stigmatization experiences, which can lead to higher minority stress levels [26] and increased self-reported disability [28]. This in turn has a negative impact on mental health [28] and especially the emergence of clinically relevant depressive [2] and anxiety disorders [29], which ultimately affects QoL.

### 4.4. Influencing and Predictive Factors

The one-way ANOVA and the *t*-test comparisons show significant associations between participants' mental QoL and certain sociodemographic factors (age, work situation, and relationship status) and GAI factors (hormone treatment and surgical measures). These findings support the investigations of Motmans et al. [7] who revealed the influence of these sociodemographic factors on QoL in female and male trans persons. Beyond that, an association between these sociodemographic factors and QoL was also outlined among the general population [40]. With regard to GAI and QoL, the positive relationship is well documented [11–14].

However, surprisingly, none of these variables had a relevant predictive impact on the mental QoL in our model. Only the categories gender and work situation had significant regression weights, but these could only explain a small fraction of the MCS variance. But neither the hormonal nor the surgical obtained any predictive significance regarding QoL. This result is, on one hand, in accordance with the findings of Gómez-Gil et al. [19] who found employment to be a (minor) predictive factor of QoL. On the other hand, this contrasts with previous work by Motmans et al. [7] who identified not only gender but also age and relationship status as influencing factors.

The reasons for these disparate observations are complex and difficult to determine. In principle, they can be related to definition, methodological, and conceptual issues. Due to the multiple changes that the trans phenomenon has undergone over time [1, 41, 42], the inclusion criteria of the respective trans populations in various studies differ greatly [13, 14, 16, 28]. This is particularly evident in the visibility of nonbinary trans persons, where the explicit coupling of inclusion and ICD-10 transsexualism definition criteria leads to automatic exclusion. In addition, many previous studies have examined trans persons regardless of their transition stage. Accordingly, a transparent comparison of the results is impossible due to these differences in definitions. The absence of a significant correlation of GAI and QoL in our model might stem from the biased narrowing of our sample. Given that being in an initial phase of transition [43] or not yet having begun planned medical GAI [44] is associated with worse mental health, the decision to include only trans persons who were actually able to carry out the interventions they wished could have led to loosening of the link between mental QoL and GAI. Furthermore, it might be that the SF-36 questionnaire does not sufficiently display the QoL concerns of trans persons. Even if it is a widely used and validated instrument on QoL [7, 11–14, 20], the SF-36 questionnaire might focus too much on irrelevant aspects of this population's wellbeing.

These difficulties raise the conceptual question: which factors actually impact the QoL of transitioned trans persons? Our results indicate that the wellbeing of trans individuals must depend on factors other than GAI. Thus, while medical interventions are crucial to the transition of trans persons, they do not define the endpoint of stabilizing QoL [45]. Here, stigmatization and its resulting gender minority stress [35] might play an essential role as further influencing factors. This is in accordance with Başar et al. [46] who claimed perceived personal discrimination to predict QoL and Bockting et al. [47] who found social stigma to be positively associated with psychological distress. In this sense, the future exploration of QoL of trans persons should consider not only the impact of medical measures but also the psychosocial consequences of the minority position of trans persons.

### 4.5. Limitations and Strengths

Our data cannot be generalized to the entire trans population. Due to our study design, only trans persons who have undergone a medical transition participated in this study. Thus, the findings cannot be applied to trans persons who decline the use of GAI. Likewise, trans persons who have not yet started a medical transition or are currently in the process of such measure were excluded. Therefore, our data does not allow any statements regarding this group of trans persons. At the same time, our inclusion criteria led to homogenization of the group, which was clearly missing in some previous studies.

A further limitation of our study is the missing information on the participants' current level of gender dysphoria. This could be an important predictive factor for the QoL of trans persons. Concurrently, the trans people enrolled here defined their medical transition as “completed.” In this sense, we can assume that no trans person who participated hoped to improve their QoL by initiating further medical measures. In addition, the cross-sectional design of this study does not allow further comparison of the QoL before the medical transition of the participants. Accordingly, no statements can be made as to whether the GAI changed the QoL of the trans persons or not. However, this study design allowed for many trans persons to be made aware of the ongoing investigation. By using two ways of recruitment and cooperating with trans organizations, we executed one of the largest surveys of trans persons in Central Europe.

## 5. Conclusions

The results emphasize that trans individuals are at greater risk of decreased QoL and increased mental health problems than the general population. We provide evidence that gender nonbinary individuals comprise a particularly vulnerable group within the trans population and have worse mental health and, for the first time, we could identify limited QoL in this group. Therefore, somatic as well as psychosocial QoL aspects should be addressed in the medical consultation and education of trans persons. Particularly important are measures that allow the nonbinary persons to identify themselves as such and to report on their specific situation.

Finally, our data show that medical GAI are a key factor in transition and are associated with better mental wellbeing. Yet, taking into account the fact that we did not find significant correlations between GAI and QoL in our sample of transitioned trans people, future research should seek to adapt the QoL concept to the specific needs of this population. In particular, the impact of other potential influencing factors such as stigma should be investigated.

## Figures and Tables

**Table 1:** Sociodemographic characteristics. ^a^One-way ANOVA. ^b^Chi-square test.

	Transfeminine	Transmasculine	Nonbinary	*p*
Age (M (SD))	51.51 (17.06)	35.95 (12.79)	42.16 (24.41)	<0.01^a^
Level of education (*n* (%))				
Primary school	3 (3.9%)	0	0	0.06^b^
Secondary school	3 (3.9%)	4 (10%)	2 (8%)	
Apprenticeship	28 (36.8%)	15 (37.5%)	3 (12%)	
A-levels	9 (11.8%)	7 (17.5%)	8 (32%)	
University/technical college	33 (43.4%)	12 (30%)	11 (44%)	
Other	0	2 (5%)	1 (4%)	
Relationship status (*n* (%))				
Single	41 (53.9%)	17 (42.5%)	11 (44%)	<0.01^b^
In a relationship	33 (43.4%)	23 (57.5%)	7 (28%)	
Other	2 (2.6%)	0	7 (28%)	
Work situation (*n* (%))				
Self-employed	15 (19.7%)	5 (12.2%)	4 (16.7%)	0.07^b^
Employed	27 (35.5%)	25 (61%)	6 (25%)	
Unemployed	15 (19.7%)	3 (7.3%)	5 (20.8%)	
Other	19 (25%)	8 (19.5%)	9 (37.5%)	
Housing situation (*n* (%))				
With family	13 (17.1%)	9 (22%)	3 (12%)	0.1^b^
With friends/shared flat	7 (9.2%)	3 (7.3%)	7 (28%)	
With partner	22 (28.9%)	13 (31.7%)	2 (8%)	
Alone	31 (40.8)	15 (36.6%)	13 (52%)	
Other	3 (3.9%)	1 (2.4%)	0	

**Table 2:** Transition-specific data. ^a^One-way ANOVA.

	Transfeminine (*n* = 77)	Transmasculine (*n* = 41)	Nonbinary (*n* = 25)	*p*
Age at coming out (M (SD))	34.8 (14.66)	22.73 (22.32)	28.04 (10.88)	<0.01^a^
First steps in transition (M (SD))	35.12 (14.23)	27.12 (10.31)	27.62 (10.91)	<0.01^a^
Current hormone therapy (*n*)	63	41	12	
Hormone therapy in years (*n*)				
0–5	22	17	6	
6–10	4	4	1	
11–20	8	1	1	
>20	6	2	0	
Overall gender-affirming surgery (*n*)	65	39	10	
Mammoplasty (*n*)	45	38	10	
Genital reconstruction (*n*)	50	12	1	

**Table 3:** Mean scores (M (SD)) of SF-36 domains and global measures and group comparison by one-way ANOVA.

	Transfeminine	Transmasculine	Nonbinary	*p*
Physical				
Physical functioning	88.16 (21.52)	94.27 (9.46)	91.25 (9.47)	0.1
Physical role	81.91 (32.81)	92.31 (20.0)	76.0 (37.14)	0.04
Bodily pain	80.57 (27.06)	82.66 (23.06)	74.16 (27.8)	0.45
General health	74.13 (22.73)	72.58 (20.09)	53.36 (26.75)	<0.01
Mental				
Vitality	56.76 (24.47)	59.87 (17.88)	40.2 (20.23)	<0.01
Social functioning	77.17 (27.3)	79.69 (28.0)	65.0 (31.66)	0.16
Mental health	72.72 (22.78)	72.11 (19.93)	52.5 (21.44)	<0.01
Emotional role	75.32 (37.62)	89.47 (25.83)	46.67 (45.13)	<0.01
Component summary				
Physical (PCS)	53.05 (8.44)	53.76 (5.88)	53.75 (7.9)	0.87
Mental (MCS)	46.37 (12.48)	49.8 (10.25)	35.18 (14.13)	<0.01

**Table 4:** *t*-test comparison of mean scores (M (SD)) of SF-36 domains between groups and the general German population.

	Transfeminine	Cis women	*p*	Transmasculine	Cis men	*p*
Physical						
Physical functioning	88.16 (21.52)	82.71 (23.17)	0.03	94.27 (9.46)	89 (20.15)	<0.01
Physical role	81.91 (32.81)	80.41 (33.02)	0.69	92.31 (20.0)	87.3 (29.62)	0.13
Bodily pain	80.57 (27.06)	75.99 (27.68)	0.14	82.66 (23.06)	82.47 (26.56)	0.96
General health	74.13 (22.73)	66.64 (19.67)	0.01	72.58 (20.09)	69.59 (20.63)	0.35
Mental						
Vitality	56.76 (24.47)	60.62 (18.47)	0.18	59.87 (17.88)	66.17 (18.01)	0.04
Social functioning	77.17 (27.3)	87.02 (18.92)	<0.01	79.69 (28.0)	90.67 (17.51)	0.02
Mental health	72.72 (22.78)	71.44 (16.29)	0.65	72.11 (19.93)	76.55 (16.06)	0.18
Emotional role	75.32 (37.62)	88.77 (26.34)	<0.01	89.47 (25.83)	92.06 (24.58)	0.54
Component summary						
Physical (PCS)	53.05 (8.44)	49.09 (10.6)	<0.01	53.76 (5.88)	51.42 (9.62)	0.03
Mental (MCS)	46.37 (12.48)	50.71 (8.39)	0.01	49.8 (10.25)	52.44 (7.7)	0.15

**Table 5:** Descriptive results of SF-36 global measures and sociodemographic data with group comparison of mean scores (M (SD)) by one-way ANOVA.

	PCS	*F* (df)	*p*	MCS	*F* (df)	*p*
Age						
18–35 years (*n* = 40)	53.22 (7.41)	2.29 (2)	0.11	41.17 (13.99)	3.02 (2)	0.05
36–50 years (*n* = 32)	55.76 (4.74)			46.61 (13.4)		
>50 years (*n* = 45)	52.04 (9.17)			46.61 (13.4)		
Level of education						
Primary school	46.94 (15.16)	1.66 (5)	0.15	43.13 (12.83)	1.14 (5)	0.34
Secondary school	50.68 (7.27)			45.90 (10.35)		
Apprenticeship	51.59 (8.98)			45.29 (13.42)		
A-levels	54.39 (5.86)			40.04 (14.75)		
University/technical college	55.14 (6.6)			47.05 (12.59)		
Other	53.79 (3.32)			54.39 (8.37)		
Relationship status						
Single	52.63 (7.32)	0.61 (2)	0.55	44.17 (14.15)	4.45 (2)	0.01
In a relationship	53.97 (7.86)			47.82 (11.61)		
Other	55.03 (9.6)			33.15 (13.17)		
Work situation						
Employed	55.5 (5.89)	6.24 (2)	<0.01	49.18 (11.06)	7.47 (2)	<0.01
Unemployed	49.94 (9.31)			40.92 (11.58)		
Other	51.57 (8.24)			40.12 (14.94)		
Housing situation						
With family	54.05 (7.19)	1.67 (4)	0.16	44.26 (10.89)	2.23 (4)	0.07
With friends/shared flat	55.45 (3.77)			39.37 (17.05)		
With partner	54.89 (7.1)			49.64 (10.73)		
Alone	51.29 (8.83)			44.01 (13.71)		
Other	57.6			59.88		

**Table 6:** Descriptive results of SF-36 global measures and transition-specific datawith *t*-test comparison of mean scores (M (SD)).

	PCS	*p*	MCS	*p*
Current hormone therapy				
Yes	54.03 (6.43)	0.19	46.76 (11.89)	0.04
No	50.94 (11.2)		39.39 (16.04)	
Overall gender-affirming surgery				
Yes	53.83 (6.69)	0.59	46.81 (12.02)	0.01
No	52.67 (10.24)		39.76 (15.33)	

**Table 7:** Block-wise multiple regression analysis displaying the predictive value of transition-specific and sociodemographic factors on mental quality of life.

Block	Predictor	MCS			
		*β*	*p*	Corrected *R*^2^	*R* ^2^ change
1	Transfeminine	.373	.005	.144	.158
	Transmasculine	.390	.005		

2	Overall gender-affirming surgery	.026	.814	.139	.002

3	Hormonal therapy	.029	.786	.135	.003

4	In a partnership	.091	.600	.135	.014
	Single	.030	.860		

5	Employment	.299	.003	.196	.071
	Unemployment	.038	.701		
